# Colorectal cancer cell-derived microvesicles are enriched in cell cycle-related mRNAs that promote proliferation of endothelial cells

**DOI:** 10.1186/1471-2164-10-556

**Published:** 2009-11-25

**Authors:** Bok Sil Hong, Ji-Hoon Cho, Hyunjung Kim, Eun-Jeong Choi, Sangchul Rho, Jongmin Kim, Ji Hyun Kim, Dong-Sic Choi, Yoon-Keun Kim, Daehee Hwang, Yong Song Gho

**Affiliations:** 1 Division of Molecular and Life Sciences, Pohang University of Science and Technology, Pohang 790-784, Republic of Korea; 2 School of Interdisciplinary Bioscience and Bioengineering, Pohang University of Science and Technology, Pohang 790-784, Republic of Korea; 3 Department of Chemical Engineering, Pohang University of Science and Technology, Pohang, Republic of Korea

## Abstract

**Background:**

Various cancer cells, including those of colorectal cancer (CRC), release microvesicles (exosomes) into surrounding tissues and peripheral circulation. These microvesicles can mediate communication between cells and affect various tumor-related processes in their target cells.

**Results:**

We present potential roles of CRC cell-derived microvesicles in tumor progression via a global comparative microvesicular and cellular transcriptomic analysis of human SW480 CRC cells. We first identified 11,327 microvesicular mRNAs involved in tumorigenesis-related processes that reflect the physiology of donor CRC cells. We then found 241 mRNAs enriched in the microvesicles above donor cell levels, of which 27 were involved in cell cycle-related processes. Network analysis revealed that most of the cell cycle-related microvesicle-enriched mRNAs were associated with M-phase activities. The integration of two mRNA datasets showed that these M-phase-related mRNAs were differentially regulated across CRC patients, suggesting their potential roles in tumor progression. Finally, we experimentally verified the network-driven hypothesis by showing a significant increase in proliferation of endothelial cells treated with the microvesicles.

**Conclusion:**

Our study demonstrates that CRC cell-derived microvesicles are enriched in cell cycle-related mRNAs that promote proliferation of endothelial cells, suggesting that microvesicles of cancer cells can be involved in tumor growth and metastasis by facilitating angiogenesis-related processes. This information will help elucidate the pathophysiological functions of tumor-derived microvesicles, and aid in the development of cancer diagnostics, including colorectal cancer.

## Background

During growth or activation, a variety of cell types, including hematopoietic, epithelial, and tumor cells, shed small membrane vesicles called microvesicles (exosomes) [1-6]. These microvesicles, which are 30-200 nm in diameter, are derived from the endosomal membrane compartment following the fusion of multivesicular bodies with the plasma membrane. Cells also release microvesicles directly by the outward budding of the plasma membrane in a calcium-dependent manner [4]. Microvesicles have been also found in various body fluids, such as plasma, malignant pleural effusion, and urine [7-10]. The numerous proteins and bioactive lipids contained in microvesicles differ in composition depending on the types and states of donor cells. Recent studies have reported that microvesicles also contain mRNA [11-15]. Although their biological roles are not completely understood, microvesicles have been shown to stimulate target cells by transferring microvesicular components (e.g., plasma membrane receptors and bioactive lipids) into target cells, transferring infectious particles, such as human immunodeficiency virus and prions, and delivering microvesicular mRNAs to target cells (called horizontal transfer of genetic materials), where they are translated into functionally active proteins [12-15]. These findings suggest that microvesicles are not cellular debris but are communicasomes, which are extracellular organelles with distinct roles in intercellular communication [5,16].

Colorectal cancer (CRC) is one of the most frequent malignant tumors in Western countries [17]. CRC cells also release microvesicles [5,18], and the revelation from proteomic studies that CRC cell-derived microvesicles contain several hundred proteins has helped elucidate the functions of microvesicles at the protein level [5,18]. It has also been reported that RNA-lipid complexes are released from the plasma membrane of CRC cells, and CRC patients show significantly elevated serum mRNA levels over those of healthy subjects [19,20]. In view of the higher concentration of RNase in the serum of patients with cancer, these circulating mRNAs are likely to be present in a particle-associated form [21,22]. Collectively, these observations suggest that CRC cell-derived microvesicles carry mRNA, together with proteins and lipids, which may exert a functional influence on CRC-related processes.

To improve the understanding of the potential roles of CRC cell-derived microvesicles in CRC-related processes, we conducted a systems approach to the CRC cell-derived microvesicles using transcriptome analysis of the microvesicles derived from SW480 cells, a human CRC cell line, and the donor CRC cells. Our systems approach to CRC cell-derived microvesicles provided 11,327 microvesicular transcripts that are involved in various tumor-related processes, which suggests that the microvesicles reflect the physiology of the donor CRC cells. Furthermore, we found that CRC-derived microvesicles enriched with cell cycle-related mRNAs that showed differential expression patterns in CRC patient data. Moreover, microvesicles stimulated proliferation of endothelial cells, suggesting that CRC-derived microvesicles can be involved in tumor growth and metastasis by facilitating angiogenesis-related processes. Our results provide a number of indicators that will not only increase the understanding of pathophysiological functions of tumor derived microvesicles, but also may stimulate the development of novel methods for cancer diagnostics including CRC.

## Results

### Purification and characterization of microvesicles derived from human SW480 cells

**Figure 1:**
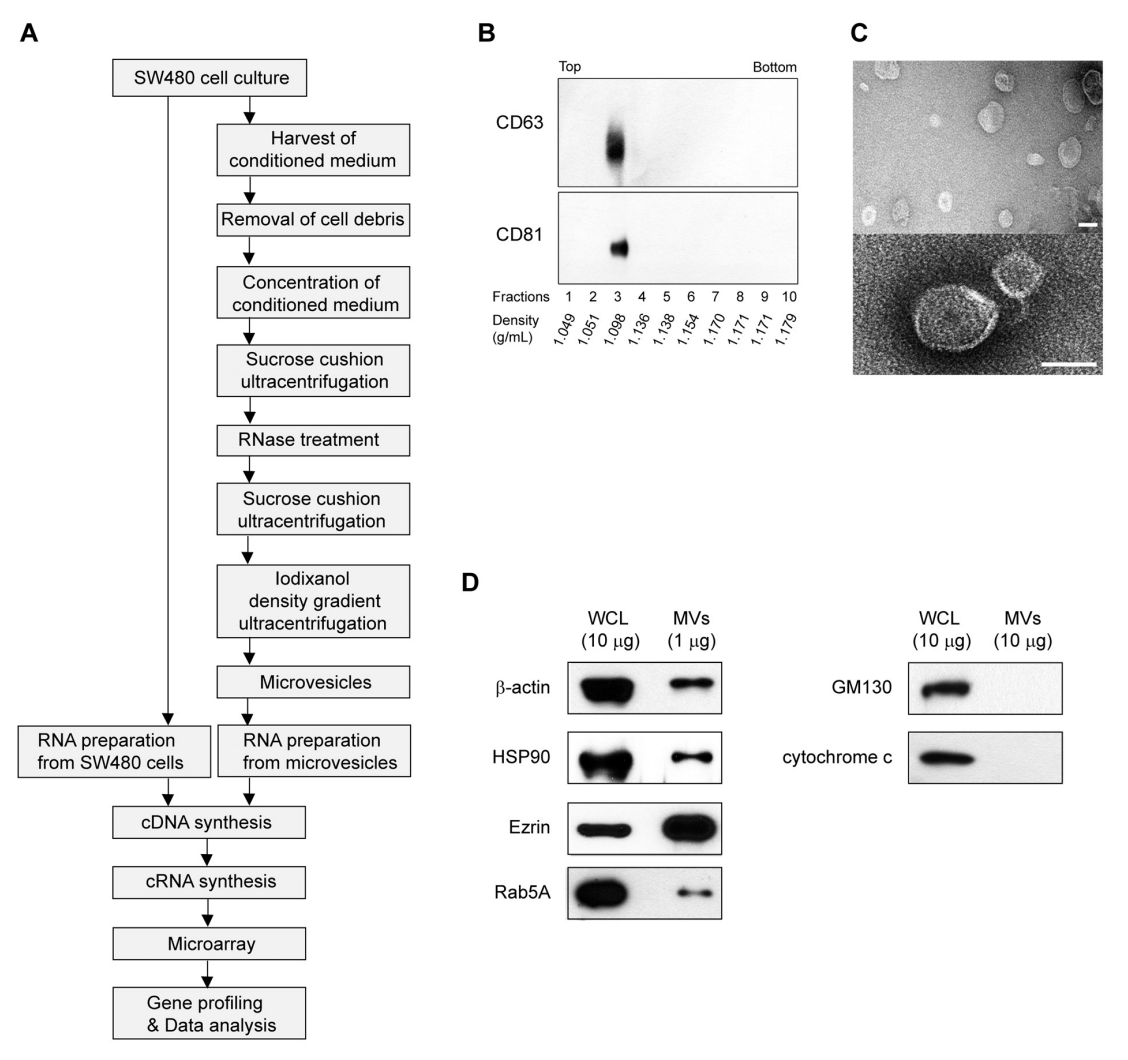
**Purification and characterization of microvesicles derived from SW480 cells**. A) Overview of the procedure used to identify microvesicular and cellular mRNA from SW480 cells. B) Western blot of microvesicular marker proteins CD63 and CD81 in fractions from iodixanol density gradients. C) Electron microscopy of the purified microvesicles. Bars represent 100 nm. D) Western blot of β-actin, HSP90, Ezrin, and Rab5A (microvesicular proteins), and GM130 (a protein found in the cis-Golgi apparatus), and cytochrome c (a mitochondrial protein found in apoptotic bodies). Neither GM130 nor cytochrome *c *were detected in microvesicles, despite using 10 times more microvesicles (10 μg) than the amount used to detect other microvesicular proteins (1 μg). The symbols of WCL and MVs represent whole cell lysate and microvesicles, respectively.

We obtained highly purified microvesicles secreted by human colorectal adenocarcinoma SW480 cells using a previously described method with several modifications (Figure 1A) [5]. Microvesicles were isolated from the culture supernatant using a combination of differential centrifugation to remove cells and cellular debris, ultrafiltration through a 100-kilodaltons hollow-fiber membrane to concentrate the microvesicles, and ultracentrifugation onto sucrose cushions. The purified microvesicles were treated with RNase A to degrade any non-microvesicular RNA and then further purified using iodixanol density-gradient ultracentrifugation to remove non-membranous proteins, protein aggregates, and denatured microvesicles. The microvesicles settled at a density of ~1.098 g/mL, as indicated by the microvesicular marker proteins CD63 and CD81 (Figure 1B). An examination of the purified microvesicles using electron microscopy revealed that nearly all were small closed vesicles, approximately 40-150 nm in size (Figure 1C) [5,18]. Furthermore, β-actin, HSP90, ezrin, and Rab5A, which are known microvesicular proteins, were detected in the purified microvesicles, whereas GM130, a protein present in the *cis*-Golgi apparatus, and cytochrome *c*, a mitochondrial protein present in apoptotic bodies, were not detected (Figure 1D) [5].

### Transcriptomic analysis of microvesicles

**Figure 2:**
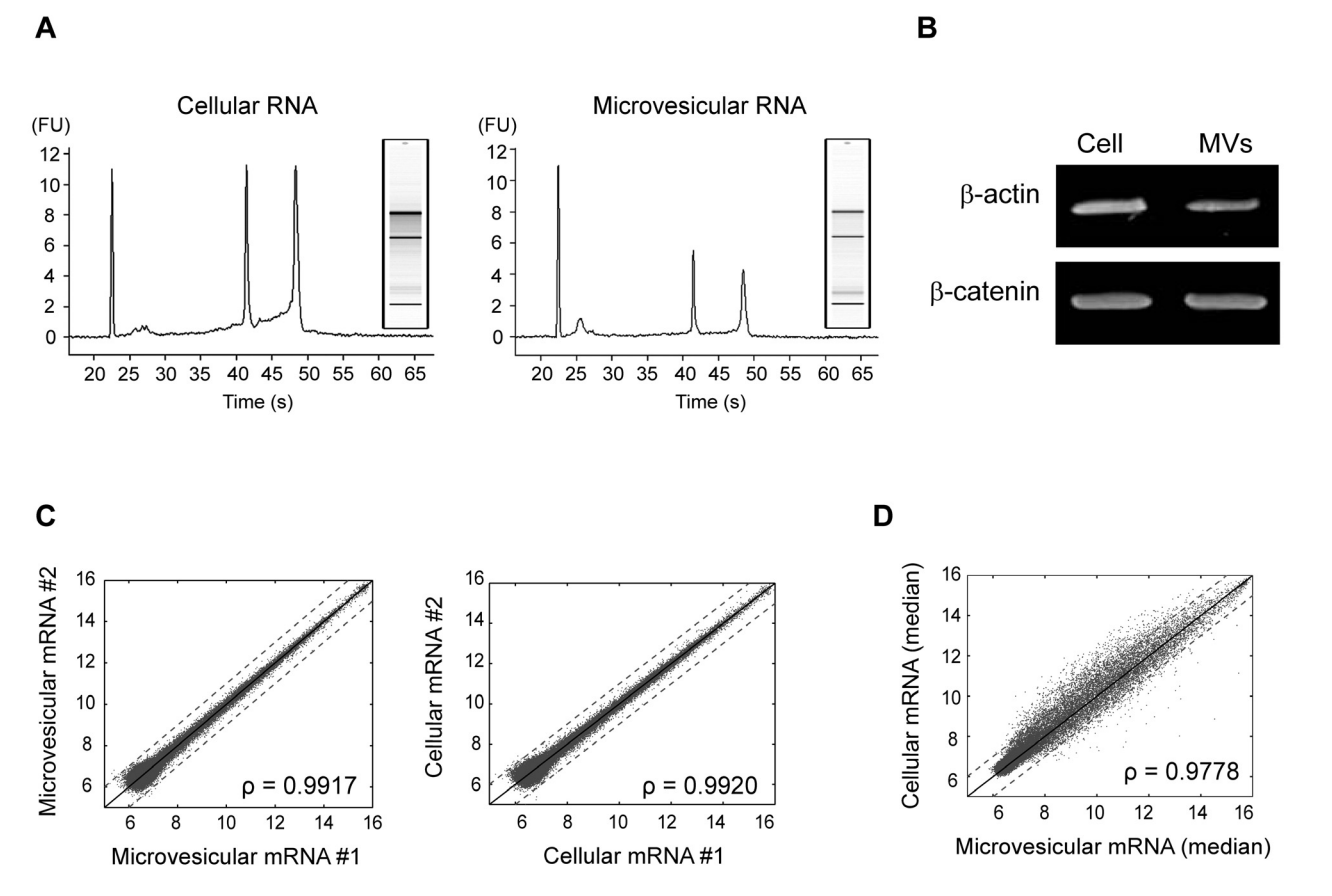
**Isolation and transcriptomic analysis of microvesicular RNA**. A) Bioanalyzer results of total RNA isolated from microvesicles and SW480 cells. The third and fourth peaks correspond to 18S and 28S rRNA, respectively. Images of gel electrophoresis are also shown. B) Gel electrophoresis of microvesicular and cellular β-actin and β-catenin mRNA amplified by RT-PCR. C) Scatterplots of microvesicular and cellular mRNA expression levels showing log2 intensities in the first and the second arrays (Pearson's correlation coefficients: ρ = 0.9917 and 0.9920 for microvesicular RNA and cellular RNA, respectively). D) Scatterplot of median log2 intensities of the four microvesicle replicates versus those of their donor cells (ρ = 0.9778).

For transcriptomic profiling, we extracted high-quality total RNA from both purified microvesicles and SW480 cells (Figure 1A), as indicated by the high RNA integrity numbers of Bioanalyzer profiles: 9.0 for cellular RNA and 8.7 for microvesicular RNA (Figure 2A). The microvesicular β-actin and β-catenin transcripts were further confirmed by RT-PCR analysis (Figure 2B). We then prepared cRNA and hybridized the cRNA to Illumina Human-6 v2 Expression BeadChips (four technical replicates each for the microvesicular and cellular mRNAs; Figure 1A). The technical replicates had a high reproducibility, as shown by the high Pearson's correlation (> 0.99) between the normalized intensities of two independent arrays of microvesicular and cellular RNAs (Figure 2C and additional file 1). Moreover, mRNA abundances from SW480 cells and microvesicles (Figure 2D) were also highly correlated (correlation coefficient = 0.9778), indicating that similar amounts of most cellular mRNAs also exist in microvesicles.

**Figure 3:**
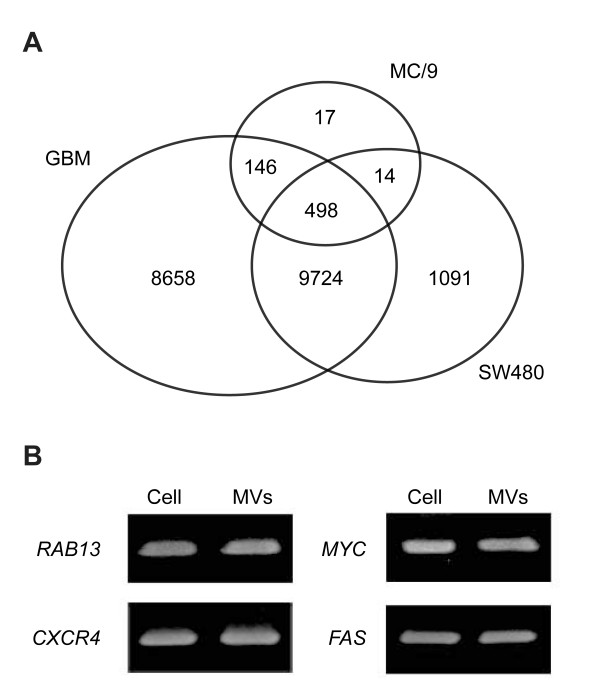
**Comparison with other microarray studies and validation of the microvesicular and cellular mRNAs detected in our study**. A) Comparison among the number of mRNAs detected in microvesicles derived from mouse mast cells (MC/9) [14], glioblastoma (GBM) [15] and human SW480 CRC cells. B) Gel electrophoresis of amplified microvesicular and cellular mRNA. Presence of several microvesicular mRNAs including *RAB13, CXCR4, MYC*, and *FAS *were confirmed using RT-PCR. The symbol of MVs represents microvesicles.

From the mRNA measurements, we found that most of the mRNA transcripts from SW480 cells (11,624) also existed in SW480-derived microvesicles (11,327) (additional file 2). This finding is similar to that of Skog et al. (2008), who detected about 27,000 transcripts in glioblastoma-derived microvesicles [15]. In contrast, two previous studies [13,14] reported that only subsets of genes in endothelial progenitor cells and mouse mast cells (MC/9) were selectively sorted into microvesicles. A comparison of microvesicular mRNAs in our study with those in two previous studies [14,15] (Figure 3A) showed that the majority of microvesicular mRNAs in those studies were also detected in our study: 10,222 overlapping transcripts from glioblastoma cells and 512 from mouse mast cell MC/9 (additional file 3). Using RT-PCR, we confirmed that among the microvesicular transcripts shared by SW480 and glioblastoma microvesicles [15] but not detected in mast cell microvesicles, *RAB13*, *CXCR4*, *MYC*, and *FAS *transcripts were present in the SW480-derived microvesicles (Figure 3B).

### Cellular processes overrepresented by the microvesicular mRNAs

**Figure 4:**
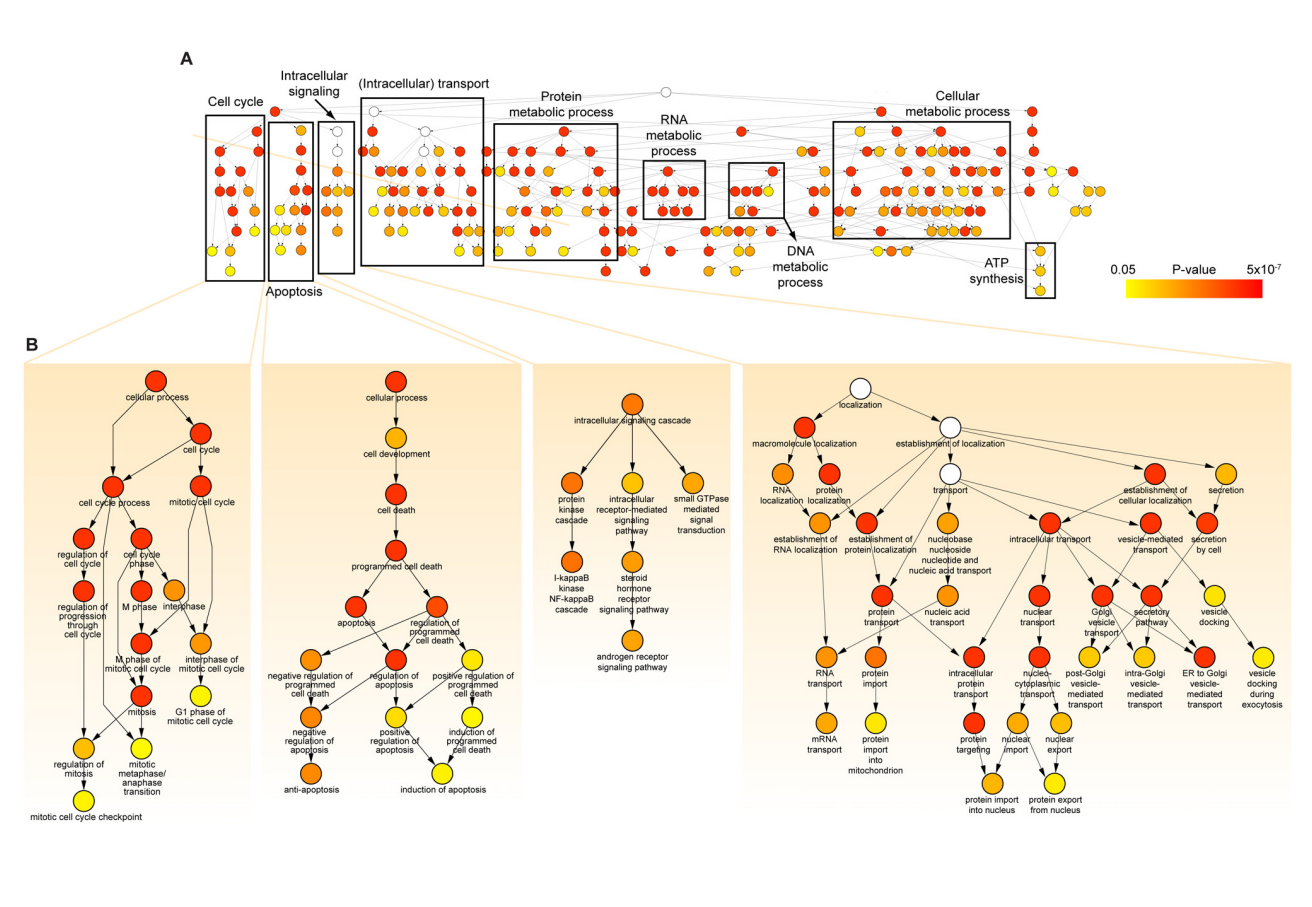
**Cellular processes overrepresented by the mRNAs detected in microvesicles**. A) A GO tree obtained from BiNGO showing the hierarchy of GOBPs overrepresented by mRNAs present in microvesicles (*P *< 0.05; see the color bar). Node colors represent the statistical significance of functional enrichment of the corresponding GOBPs. The white nodes (*P *> 0.05) were added to show the relationships among their downstream nodes. The overrepresented GOBPs were categorized into several groups of cellular processes, each of which is indicated by a box. B) The four GOBP groups (cell cycle, apoptosis, intracellular signaling, and intracellular transport) related to tumor progression are shown in detail.

We then identified functions overrepresented by the 11,327 microvesicular mRNAs using BiNGO, a Cytoscape plugin [23]. Figure 4A shows a GO tree representing a hierarchical structure of GOBPs, in which the colored nodes in the GO tree indicate significantly overrepresented GOBPs (*P *< 0.05). The overrepresented cellular processes were categorized into the following groups: cell cycle, cell death, intracellular signaling, intracellular transport, and metabolic processes of protein, RNA, and DNA (boxes in Figure 4A). Among these groups, Figure 4B shows the four groups of GOBPs closely associated with tumor progression: cell cycle- and mitosis-related processes, apoptosis-related processes, tumor-related intracellular signaling pathways, and vesicle-mediated transport pathways that might be involved in the formation and secretion of microvesicles. Collectively, these observations suggest that microvesicular mRNAs probably can contribute to promoting tumor progression in target cells when these mRNAs are transferred into neighboring target cells.

### Enrichment of cell cycle-related mRNAs in microvesicles

**Figure 5:**
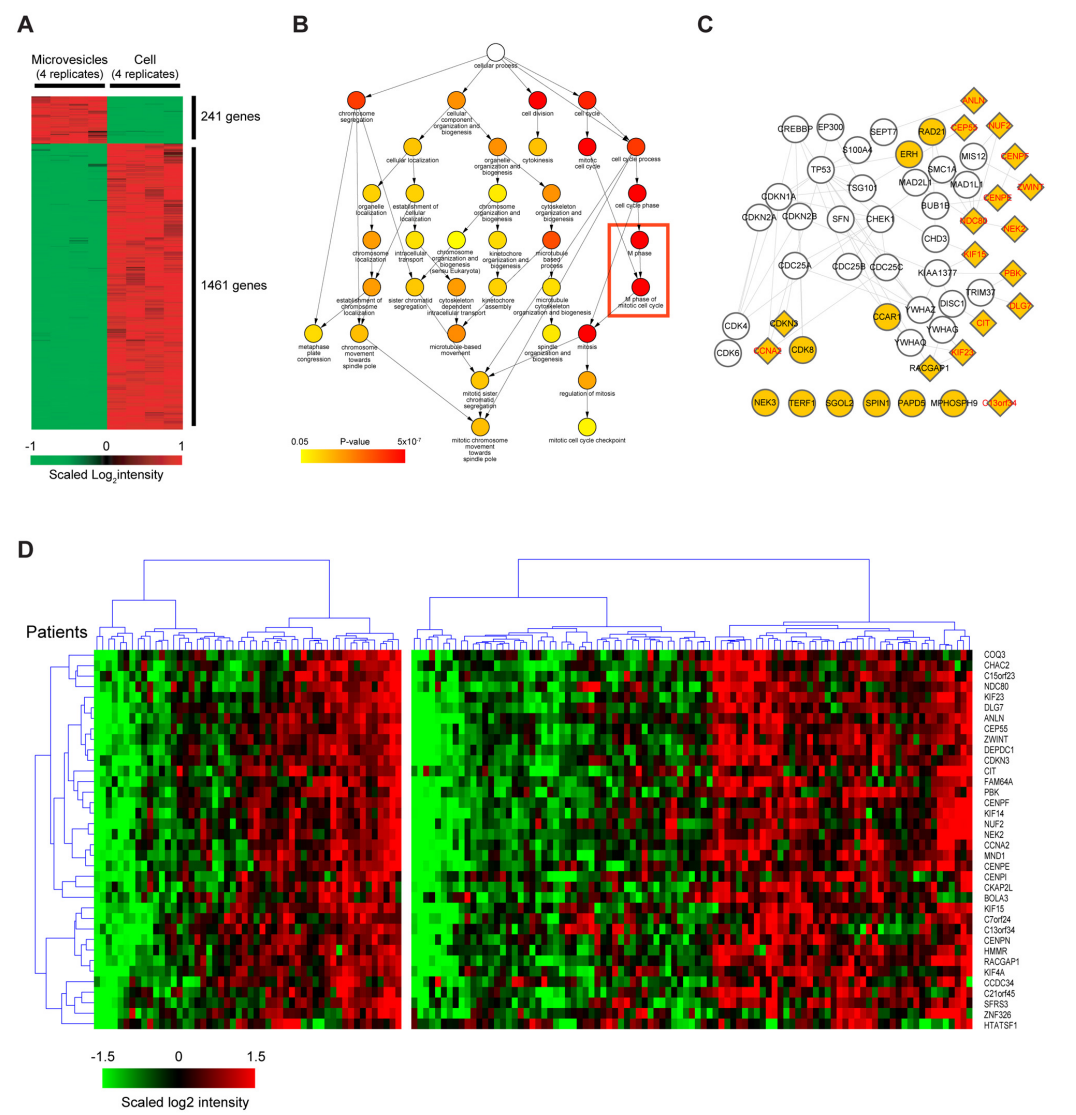
**A biological network describing the cellular processes overrepresented by microvesicle-enriched mRNAs**. A) A heat map illustrating the expression patterns of the 1,702 genes that exhibited a more than 2-fold difference in the mRNA level between microvesicles and cells. For clarity, the expression of each is scaled to a mean of zero with unit variance across all eight replicates. The colors represent the relative increase (red) or decrease (green) in the expression level of the corresponding gene in microvesicles compared with the level in cells: 241 and 1,461 genes were overexpressed in microvesicles and in cells, respectively. B) A GO subtree showing cell cycle-related GOBPs overrepresented by the 241 microvesicle-enriched genes. The color scheme of Figure 4 is repeated. Notably, cell cycle-related GOBPs were strongly overrepresented by 27 mRNAs, as indicated by the dark-red node colors (see text for details). C) A hypothetical network reconstructed using the 27 microvesicle-enriched mRNAs belonging to the cell cycle-related GOBPs in (B) and their first and second interaction neighbors obtained from protein-protein interaction databases (see Materials and methods). The yellow nodes represent the 27 cell cycle-related microvesicle-enriched genes: 20 of the 27 genes closely interact with cell cycle-related processes. The lines between the nodes represent protein-protein interactions. The network shows that the microvesicle-enriched genes closely interact with key pathways in the cell cycle. D) Cluster heat map comprising 36 of the 241 microvesicle-enriched mRNAs showing consistent differential expression patterns across patients in two independent CRC datasets (GSE2109 and GSE5206). The diamond nodes in the network (C) represent the 17 nodes shared by the 27 cell cycle-associated mRNAs and 36 mRNAs; of the 17 nodes, 15 (labeled in red) are associated with M-phase-related processes.

Despite the similarities at the global level between the microvesicular and cellular mRNAs, we identified 241 mRNAs whose expression levels were two fold higher in the microvesicles than in SW480 cells (microvesicle-enriched mRNAs; Figure 5A and additional file 4). We also identified 1,461 mRNAs whose expression levels were two fold higher in SW480 cells than in microvesicles (cell-enriched mRNAs). To characterize the processes overrepresented by the microvesicle-enriched genes, we performed GO enrichment analysis using BiNGO, where among the 241 microvesicle-enriched genes, 136 genes with GO annotations were used (additional file 5). Unexpectedly, cell cycle-related processes (GO term "cell cycle"), including M-phase and mitosis (dark red nodes in Figure 5B), are highly overrepresented by 27 out of 136 microvesicle-enriched genes (*P *= 2.98 × 10^-8^). Moreover, 20 of these 27 genes also belong to M-phase cell cycle processes (red box in Figure 5B, *P *= 6.27 × 10^-13^).

To predict the potential roles of the 27 cell cycle-related microvesicle-enriched genes at the molecular level, we then reconstructed a biological network (Figure 5C) describing the overrepresented cell cycle-related processes (Figure 5D). The network was reconstructed using the 27 genes belonging to the cell cycle GO term and their first and second interaction neighbors obtained from HPRD, BIND, and BioGRID interaction databases. In the network, yellow nodes represent the 27 microvesicle-enriched genes, 20 of which closely interact with cell cycle-related processes. The network shows that the microvesicle-enriched genes closely interact with key pathways in the cell cycle, including cyclin-dependent kinases 8 and N3 (*CDK8 *and *CDKN3*), centrosomal protein 55 kilodaltons (*CEP55*), kinensin family members (*KIF15 *and *KIF23*), centromere proteins (*CENPE *and *CENPF*), anillin (*ANLN*), PDZ-binding kinase (*PBK*), and NIMA-related kinase 2 (*NEK2*). Collectively, these observations suggest that the microvesicle-enriched genes may contribute to the increased cell proliferation of target cells during tumor progression by modulating activities of cell cycle-related pathways.

To examine the clinical implications of the 241 microvesicle-enriched genes, we collected from the GEO database [24] two transcriptomic datasets (GSE2109 and GSE5206), including global mRNA measurements from CRC patients at various stages, and then used cluster analysis to investigate whether these genes are differentially expressed among patients (additional file 6). Interestingly, a cluster comprising 36 of the 241 microvesicle-enriched genes showed consistent differential expression patterns across the patients in the two independent datasets (Figure 5D): the expression levels of the genes in each patient sample were compared to their average expression levels in the individual datasets. Of these, 15 of the 36 genes showing differential expression patterns are involved in M-phase-related processes (red box in Figure 5B; e.g., spindle formation, kinetochore assembly, chromosome segregation, and cytokinesis) that are either involved in or interact with components involved in the M-phase of the mitotic cell cycle (see diamond nodes in Figure 5C and additional file 7). Note that 17 of the 36 genes belong to cell cycle GOBP. These observations suggest that microvesicle-enriched genes reflect their potential roles in tumor progression in patients with CRC.

### Stimulation of endothelial cell proliferation by microvesicles

**Figure 6:**
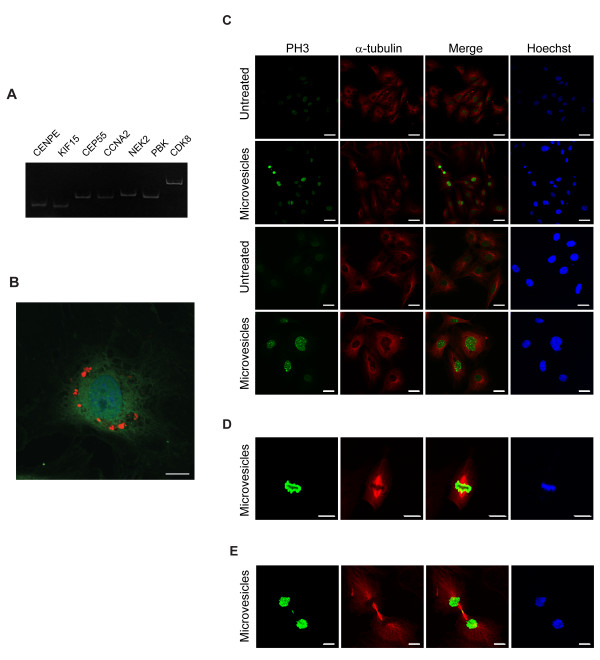
**Stimulation of endothelial cell proliferation by microvesicles**. A) Gel electrophoresis of amplified microvesicular M-phase-related transcripts. Presence of *CENPE*, *KIF15*, *CEP55*, *CCNA2*, *NEK2*, *PBK *and *CDK8 *were confirmed using RT-PCR. B) Immunofluorescence of HUVECs treated with microvesicles labeled with PKH26 (red). Cells were stained with anti-calnexin antibody, an endoplasmic reticulum marker protein (green). C-E) Immunostaining of HUVECs with anti-phospho-histone H3 (green) and anti-α-tubulin antibodies (red). After 12 hours, microvesicle-treated endothelial cells were at metaphase (D) and cytokinesis with mitotic spindle formation (E). None of the untreated control endothelial cells were in metaphase or cytokinesis stages. Scale bars represent 10 μm (B, D, E) and 40 μm (C). The symbol of PH3 represents phospho-histone H3.

Based on the observations that microvesicle-enriched mRNAs are highly associated with M-phase-related processes (Figure 5), we generated a network-driven hypothesis that CRC cell-derived microvesicles promote the proliferation of neighboring cells in the tumor microenvironment by increasing their cell cycle activities via the horizontal transfer of microvesicular mRNAs, as several recent studies have clearly demonstrated [12-15]. To experimentally verify this hypothesis, we investigated whether CRC cell-derived microvesicles can affect the cell cycle of endothelial cells. Using RT-PCR, we first confirmed that M-phase-related transcripts including *CENPE*, *KIF15*, *CEP55*, *CCNA2*, *NEK2*, *PBK*, and *CDK8 *were present in the SW480-derived microvesicles (Figure 6A). When we incubated microvesicles labeled with fluorescent dye with HUVECs, we observed a strong red fluorescence in the cytoplasm (Figure 6B), indicating that significant amounts of microvesicles were taken up by the HUVECs. We then immunostained both phospho-histone H3 (mitosis marker) and α-tubulin to analyze mitotic spindle formation occurring during the M-phase. A subpopulation of microvesicle-treated HUVECs, indicated by a strong phospho-histone H3 signal, underwent mitosis (Figure 6C). Furthermore, at higher magnification, we observed these microvesicle-treated HUVECs to be at metaphase (Figure 6D), a stage of M-phase, and undergoing cytokinesis with mitotic spindle formation (Figure 6E). In contrast, HUVECs not treated with microvesicles showed weak histone H3 phosphorylation (Figure 6C), and none were at metaphase/cytokinesis stages (data not shown). Furthermore, we observed that HUVECs treated with microvesicles derived from THP-1, human acute monocytic leukemia cell line showed weak histone H3 phosphorylation (additional file 8). These observations clearly support a role for CRC cell-derived microvesicles in modulating M-phase activities of the cell cycle and thus promoting proliferation of HUVECs, which can initiate angiogenesis.

## Discussion

This study presents a transcriptomic analysis of CRC cell-derived microvesicles, among which we identified 11,327 microvesicular transcripts involved in various tumor-related processes that reflect the physiology of the donor CRC cells. Among these transcripts, we found 241 mRNAs enriched in microvesicles beyond their levels in donor cells, of which 27 were involved in the cell cycle. Moreover, 20 of these 27 mRNAs were associated with cell cycle M-phase activities, and 15 of these showed differential expression patterns in CRC patient data. We then demonstrated that these mRNAs may modulate activities of M-phase processes during the cell cycle by reconstructing a cell cycle network, which showed that the corresponding proteins belong to and also interact with those involved in M-phase-related processes. To experimentally verify this network-driven hypothesis, we demonstrated that CRC cell-derived microvesicles are delivered to endothelial cells and stimulate the proliferation of endothelial cells. Since this proliferation is an essential step in neovascularization, our finding suggests that microvesicles have angiogenic activities.

The preparation of microvesicles that are not contaminated with cellular debris, bovine serum-derived microvesicles, or any non-microvesicular mRNA is critical for transcriptional profiling of microvesicles. As the half-life of individual microvesicular mRNA transcripts is unknown, the rapid analysis of microvesicular mRNA is also very important. We therefore followed our previously described purification methods with the following two major modifications [5]. In particular, RNase A treatment was applied in order to degrade non-microvesicular RNA derived from damaged cells or leaky microvesicles (Figure 1A). The microvesicles were finally purified using iodixanol density gradient centrifugation, which is much faster than the conventional sucrose density gradient centrifugation [14,25]. As a result, the high quality of the microvesicular RNA was obtained as indicated by the Bioanalyzer results (RIN: 8.7).

Growing evidence indicates that tumor-derived microvesicles are involved in tumor progression via the modulation of tumorigenesis, angiogenesis, immune response, invasion, and metastasis [1-5]. Moreover, recent studies have reported that microvesicular mRNA can induce potent epigenetic changes in target cells, as microvesicular mRNA is delivered to target cells, where it is translated into functionally active proteins [11-14]. Our findings that SW480-derived microvesicular mRNAs are involved in various tumor-related processes (Figure 4 and Figure 5) and SW480-derived microvesicles can initiate angiogenesis via the stimulation of endothelial cell proliferation (Figure 6) also suggest that the microvesicular mRNAs play potential roles in progression of CRC. Furthermore, SW480-derived microvesicles are enriched with *CENPE*, *KIF15*, *CEP55*, *CCNA2*, *NEK2*, *PBK*, and *CDK8 *(Figure 6a) that are M-phase-related transcripts involved in tumorigenesis and tumor progression [26-28]. Although we could not exclude the other possibility, our observations suggest that SW480-derived microvesicles promote the proliferation of endothelial cells by increasing their cell cycle activities via the horizontal transfer of these M-phase-related microvesicular mRNAs. Further studies on microvesicles derived from other colon cancer cells would provide valuable data. Thus, the inhibition of either microvesicle shedding or modulation of microvesicular function has been proposed as worthwhile approaches to cancer therapy. Recently, Al-Nedawi et al. (2009) showed that the treatment of Diannexin, which inhibits the uptake of the A431 (human squamous cell carcinoma cell line)-derived microvesicles into endothelial cells, to A431 tumor xenografts in mice led to a reduction of tumor growth rate and microvascular density [29]. Thus, our findings above suggest that the inhibition of biogenesis, trafficking or function of the CRC-derived microvesicles also can offer a novel therapeutic approach for CRC.

Despite the progress in understanding and treating CRC, it remains a leading cause of mortality worldwide [30]. A significant improvement in survival would be expected with early diagnosis of CRC. Several studies have reported that the level of tumor-derived microvesicles is elevated significantly in the serum or the ascites fluid of cancer patients [8,31,32]. Recently, Taylor *et al*. proposed that microRNA profiling of circulating tumor microvesicles could potentially be used to identify diagnostic markers for human ovarian cancer [33]. Thus, our finding that the microvesicular mRNA reflects the mRNA signature of the parental CRC suggests that the microvesicular mRNA may be a useful diagnostic and/or prognostic marker of early stage cancer and could be evaluated in a non-invasive manner. Possible CRC marker candidates can be predicted as the microvesicle-enriched mRNAs that show colon-specific expression patterns (additional file 9).

## Conclusion

These study present potential roles of CRC cell-derived microvesicles in tumor progression via a global comparative microvesicular and cellular transcriptomic analysis of human SW480 CRC cells. However, it is not clear how these microvesicular mRNAs cooperatively act with other microvesicular components such as lipids and proteins in the target cells. Future studies on collective pathophysiological roles of such microvesicular components in modulating dynamically tumor-related networks in the target cells will be valuable in clarifying the microvesicle-involved communication, including that in human CRC, and further unraveling the complexity of the intercellular communication involved in tumorigenesis, angiogenesis, and metastasis.

## Methods

### Cell cultures

SW480 (human colorectal adenocarcinoma cells) and THP-1 (human acute monocytic leukemia cells) were cultured in RPMI-1640 medium (Invitrogen Corporation, Carlsbad, CA) supplemented with 10% heat-inactivated fetal bovine serum (Invitrogen Corporation), 100 U/mL penicillin, and 0.1 mg/ml streptomycin. human umbilical vein endothelial cells (HUVECs) were isolated from freshly delivered umbilical cords and maintained as described previously [2].

### Purification of microvesicles

The SW480 cells were washed twice with PBS and then incubated in serum-free RPMI-1640. After 24 h incubation, the conditioned medium was collected, centrifuged at 500 × *g *for 10 minutes, and then centrifuged twice at 2,000 × *g *for 15 minutes. The supernatant was concentrated using a QuixStand Benchtop System (GE Healthcare, Bucks, UK) with a 100-kilodaltons hollow fiber membrane (GE Healthcare), placed upon 0.5 mL of 0.8 and 2.0 M sucrose cushions in buffer (20 mM HEPES, 150 mM NaCl, pH 7.4), and then centrifuged at 100,000 × *g *for 2 hours. The microvesicles were harvested from the interface between the 0.8 and 2.0 M sucrose cushions. After treatment with 20 μg/ml RNase A (Roche Diagnostics, Mannheim, Germany) for 30 minutes at room temperature, the sample was diluted 10-fold in PBS, placed upon 0.35 ml of 0.8 M and 0.15 ml of 2.0 M sucrose cushions, and centrifuged at 100,000 × *g *for 2 hours. The microvesicles were harvested and mixed with an equal volume of 60% iodixanol solution (Axis-Shield PoC AS, Oslo, Norway), to give 30% iodixanol. This sample was placed at the bottom of a tube and overlaid with 20% and 5% iodixanol. After centrifugation at 200,000 × *g *for 2 hours, 10 fractions of equal volume were removed from the top of the gradient. The microvesicle-enriched fraction was diluted with 9 ml of PBS and then centrifuged at 100,000 × *g *for 2 hours. Finally, the purified microvesicles were resuspended in PBS and used immediately for the preparation of microvesicular RNA.

### Transmission electron microscopy

The purified microvesicles were applied to glow-discharged carbon-coated copper grids (EMS, Matfield, PA). After allowing the microvesicles to absorb for 3 minutes, the grids were rinsed with droplets of de-ionized water and positive-stained with a mixture of 2% methylcellulose and 4% uranylacetate (Ted Pella, Redding, CA). Electron micrographs were recorded using a JEM 1011 microscope (Jeol, Japan) at an acceleration voltage of 100 kilovolt.

### Western blot

Whole cell lysate and microvesicles were separated by SDS-PAGE and then transferred to a polyvinylidene defluoride membrane. The membrane was blocked, incubated with antibodies followed by secondary antibodies conjugated to horseradish peroxidase and subjected to enhanced chemiluminescence.

### Preparation of total RNA and reverse-transcription polymerase chain reaction

Total RNA was extracted from SW480 cells and purified microvesicles by a single-step isolation method using TRIzol reagent (Invitrogen Corporation). The RNA was re-suspended in diethyl pyrocarbonate-treated water and quantified by UV absorbance at 260/280 nm. Reverse-transcription polymerase chain reaction (RT-PCR) was performed with 100 ng of total RNA and 100 nM forward and reverse primers, using a One Step SYBR RT-PCR kit (Takara, Otsu, Japan). The primer sequences are provided in additional file 10. A LightCycler system (Roche Diagnostics, Mannheim, Germany) was used with the following thermal profile: initial denaturation at 95°C for 10 minutes, followed by 45 cycles of 95°C for 15 seconds, 60°C for 15 seconds, and 72°C for 10 seconds.

### Microarray analysis

For transcriptional profiling, we used Illumina Human-6 v2 Expression BeadChips (Illumina, San Diego, CA), which include a bead pool of ~48,000 unique bead types corresponding to 47,294 transcripts [13]. The quality of the cellular and microvesicular total RNA was analyzed using an Agilent 2100 Bioanalyzer. 640 ng of total RNA from the microvesicles and SW480 cells were reverse-transcribed and amplified, according to the protocols in the Illumina TotalPrep RNA amplification kit manual (Ambion, Austin, TX). Then, *in vitro *transcription was carried out to generate complementary RNAs. 1.5 μg of complementary RNAs was hybridized onto each array (four replicates for the microvesicles and SW480 cells) and then labeled with Cy3-streptavidin (Amersham Biosciences, Little Chalfont, UK). The array was then scanned using a Bead Station (Illumina). The full dataset was submitted to Gene Expression Omnibus (GEO) under submission number GSE9589. The probe intensities for the microvesicles and SW480 cells were separately normalized using quantile normalization in beadarray 1.6, an R/Bioconductor package [34]. The probes were annotated using lumi 1.4, an R/Bioconductor package. To determine present probes, we applied the following Gaussian mixture modeling method [35]; 1) two Gaussian probability density functions, one for the absent probes and the other for the present ones, were fitted to the distribution of the observed probe intensity; and 2) the probes whose maximum intensities in all four replicates were higher than the threshold intensity (α: the intensity where two fitted Gaussian probability density functions meet) were determined to be present. Also, the microvesicles-enriched mRNAs were identified as the ones showing more than 2-fold changes between the median intensities of four replicates in microvesicles than those in SW480 cells. For a comparison of the microvesicular mRNAs in this study with those from the previous two studies of glioblastoma- and mouse mast cell (MC/9)-derived microvesicles [13,14], we used Affymetrix/Illumina annotation information (GPL1261, GEO accession), human-WG6 annotation, and human and mouse orthology from Mouse Genome Informatics (MGI 4.0, http://www.informatics.jax.org).

### Functional enrichment analysis and network analysis

We used BiNGO version 2.0, a Cytoscape plug-in [23], to identify biological processes overrepresented by the mRNAs detected in the microvesicles (also by the the microvesicle-enriched mRNAs). In a Gene Ontology (GO) tree, each node and node color represent the corresponding enriched Gene Ontology Biological Processes (GOBP) and the associated statistical significance (*P *values computed from a hypergeometric hypothesis test in BiNGO), respectively. To describe the cell cycle related processes in which the microvesicle-enriched mRNAs are involved and the interactions among the proteins corresponding to such mRNAs, we reconstructed an initial biological network using the proteins and their first and second neighbors [36], obtained from BIND http://www.bind.ca, HPRD http://hprd.org, and BioGrid http://www.thebiogrid.org databases, to include as many as the 27 cell cycle related microvesicle-enriched genes. To generate a compact network (Figure 4B) from the complex initial network, we then removed the nodes that were not connected with the 27 microvesicle-enriched genes, but kept the nodes whose removal would disconnect the 27 microvesicle-enriched genes from the main network.

### PKH26-labeled microvesicles

Purified microvesicles were labeled with PKH26 red fluorescent labeling kit (Sigma-Aldrich, St. Louis, MO) according to the manufacturer's instructions. The labeled microvesicles (1 μg/ml) were allowed to bind for 1 hour at 4°C, washed with PBS, and then incubated at 37°C for 24 hours. Cells were fixed with 4% paraformaldehyde, washed, and stained with calnexin (endoplasmic reticulum marker protein). The uptake of labeled microvesicles by HUVECs was determined using an FV1000 confocal microscope (Olympus, Tokyo, Japan) equipped with a UPlanSApo 40×/0.75 objective lens of red fluorescence intensity.

### Immunocytochemistry

HUVECs grown on 0.1% gelatin-coated glass coverslips were incubated with microvesicles (1 μg/ml) for the indicated times. The cells were fixed with 4% paraformaldehyde, permeablilized with 0.2% Triton X-100, and then incubated with anti-phospho-histone H3 (Ser 10) (Upstate Biotechnology, Lake Placid, NY) and anti-α-tubulin antibodies (Sigma-Aldrich). After treatment with AlexaFluor-conjugated secondary antibodies (Invitrogen Corporation), the cells were counterstained with Hoechst (Sigma-Aldrich). The FV1000 Olympus confocal microscope was used for the analysis.

### Accession numbers

The full dataset is submitted to Gene Expression Omnibus under submission number series, GSE9589.

## Abbreviations

CRC: colorectal cancer; HUVECs: human umbilical vein endothelial cells; RT-PCR: reverse-transcription polymerase chain reaction; GEO: Gene Expression Omnibus; GO: gene ontology; GOBPs: gene ontology biological processes; rRNA: ribosomal RNA.

## Authors' contributions

BSH and HK carried out sample preparation and experimental validation. JHC carried out microarray and network analysis. YSG and DH conceived of the study, and participated in its design and coordination and wrote the manuscript. All authors provided comments on the paper, and read and approved the final version of manuscript.

## Supplementary Material

Additional file 1 **Scatterplots of microvesicular and cellular mRNA**. Four replicate arrays were performed. Two arrays among #1, 2, 3, and 4 are shown in each scatterplot. The *x*- and *y*-axes represent probe intensity (log2). ρ indicates the correlation coefficient between two arrays used in each scatterplot. Click here for file

Additional file 2 **mRNAs identified as present in microvesicles and cells**. The table shows mRNAs identified as present in (A) SW480 cell-derived microvesicles and (B) SW480 cells using the Gaussian mixture modeling method. Click here for file

Additional file 3 **A comparison of microvesicular mRNAs in our study with those in two previous studies**. The table shows microvesicular mRNAs detected in 1) MC/9-derived microvesicles from Valadi et al[1], 2) glioblastoma (GBM)-derived microvesicles from Skog et al[2], and 3) SW480 cell-derived microvesicles from our study. For MC/9 data, MGI 4.0 human-mouse orthology was used to map mouse genes into their human orthologous genes http://www.informatics.jax.org. Click here for file

Additional file 4 **Two hundred and forty-one microvesicle-enriched mRNAs**. The table shows 241 mRNAs whose expression levels were two fold higher in the microvesicles than in SW480 cells. Click here for file

Additional file 5 **GOBPs overrepresented by the 241 microvesicle-enriched mRNAs**. The table shows GOBPs overrepresented by the 241 microvesicle-enriched mRNAs, 136 genes with GO annotations were used. Click here for file

Additional file 6 **A heat map showing expression patterns of the 241 microvesicle-enriched mRNAs in two independent colorectal cancer data sets (GSE2109 and GSE5206)**. Note that a cluster comprising 36 mRNAs, denoted by the box, showed consistent differential expression patterns across the patients in the two data sets. Of these, 15 are associated with M-phase-related cell cycle processes (see text for details). Click here for file

Additional file 7 **Various mRNA subsets described in the text**. The table shows various mRNA subsets of the 27 cell-cycle-associated microvesicle-enriched mRNAs and 36 mRNAs identified from the patients with CRC described in the text. Click here for file

Additional file 8 **The effect of THP-1-derived microvesicles on endothelial cell proliferation**. After 12 hours, microvesicle-treated endothelial cells were immunostained with anti-phospho-histone H3 (green) and anti-α-antibodies (red). Scale bars represent 40 μm. Click here for file

Additional file 9 **A subset of the 15 genes (*CCNA2*, *CDKN3*, *CENPF*, *KIF23*, and *NEK2*) whose expression is relatively enriched in CRC compared to other tissues and cells**. Note that the 15 genes are those shared between 20 M-phase-related genes and 36 genes with differential expression patterns in patients with CRC. Click here for file

Additional file 10 **Specific primer pairs for RT-PCR**. The table shows the primers used in this study. Click here for file
