# Supplementary material for: Colorectal cancer cell-derived microvesicles are enriched in cell cycle-related mRNAs that promote proliferation of endothelial cells
Source: BMC Genomics. 2009 Nov 25;10:556. doi: 10.1186/1471-2164-10-556 (PMC2788585; doi:10.1186/1471-2164-10-556)
Supplement: Additional file 1 — Scatterplots of microvesicular and cellular mRNA. Four replicate arrays were performed. Two arrays among #1, 2, 3, and 4 are shown in each scatterplot. The x- and y-axes represent probe intensity (log2). ρ indicates the correlation coefficient between two arrays used in each scatterplot. [file 1471-2164-10-556-S1.DOC]

**Additional file 1:** Scatterplots of microvesicular and cellular mRNA. Four replicate arrays were performed. Two arrays among #1, 2, 3, and 4 are shown in each scatterplot. The *x*- and *y*-axes represent probe intensity (log2). ρ indicates the correlation coefficient between two arrays used in each scatterplot.

**
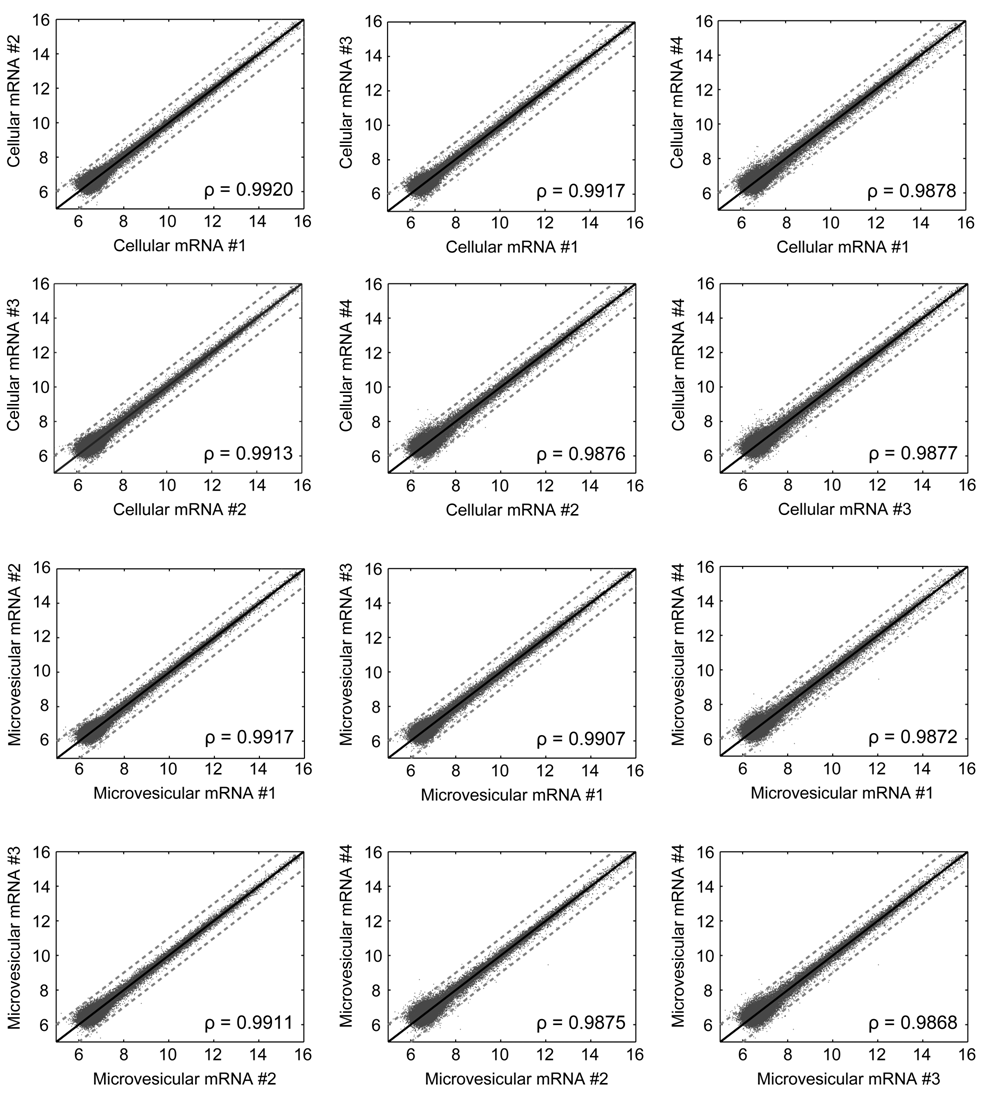
**
