# Supplementary material for: Colorectal cancer cell-derived microvesicles are enriched in cell cycle-related mRNAs that promote proliferation of endothelial cells
Source: BMC Genomics. 2009 Nov 25;10:556. doi: 10.1186/1471-2164-10-556 (PMC2788585; doi:10.1186/1471-2164-10-556)
Supplement: Additional file 6 — A heat map showing expression patterns of the 241 microvesicle-enriched mRNAs in two independent colorectal cancer data sets (GSE2109 and GSE5206). Note that a cluster comprising 36 mRNAs, denoted by the box, showed consistent differential expression patterns across the patients in the two data sets. Of these, 15 are associated with M-phase-related cell cycle processes (see text for details). [file 1471-2164-10-556-S6.DOC]

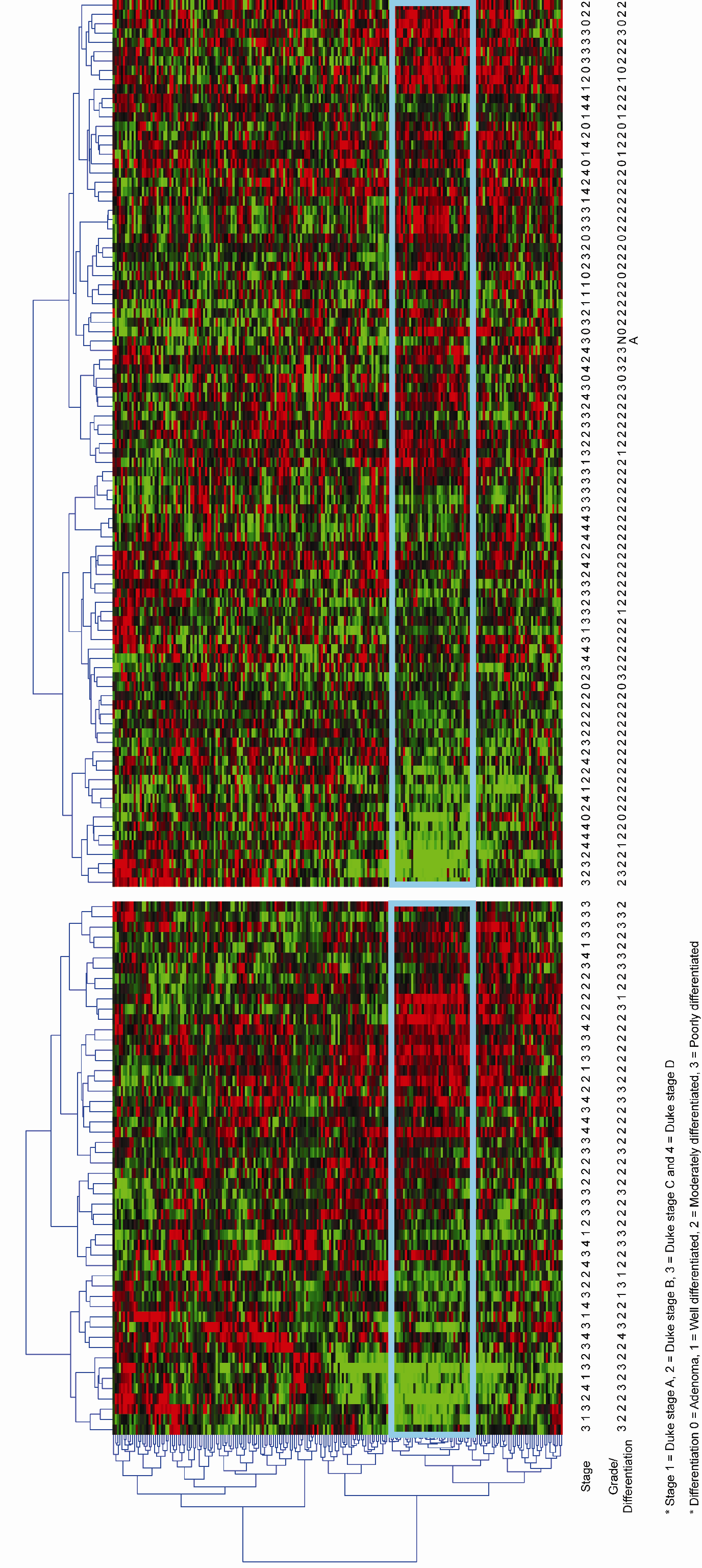
**Additional file 6.** A heat map showing expression patterns of the 241 microvesicle-enriched mRNAs in two independent colorectal cancer data sets (GSE2109 and GSE5206). Note that a cluster comprising 36 mRNAs, denoted by the box, showed consistent differential expression patterns across the patients in the two data sets. Of these, 15 are associated with M-phase-related cell cycle processes (see text for details).
