# Supplementary material for: Colorectal cancer cell-derived microvesicles are enriched in cell cycle-related mRNAs that promote proliferation of endothelial cells
Source: BMC Genomics. 2009 Nov 25;10:556. doi: 10.1186/1471-2164-10-556 (PMC2788585; doi:10.1186/1471-2164-10-556)

**Additional file 8.** The effect of THP-1-derived microvesicles on endothelial cell proliferation. After 12 hours, microvesicle-treated endothelial cells were immunostained with anti-phospho-histone H3 (green) and anti-α-antibodies (red). Scale bars represent 40 µm.


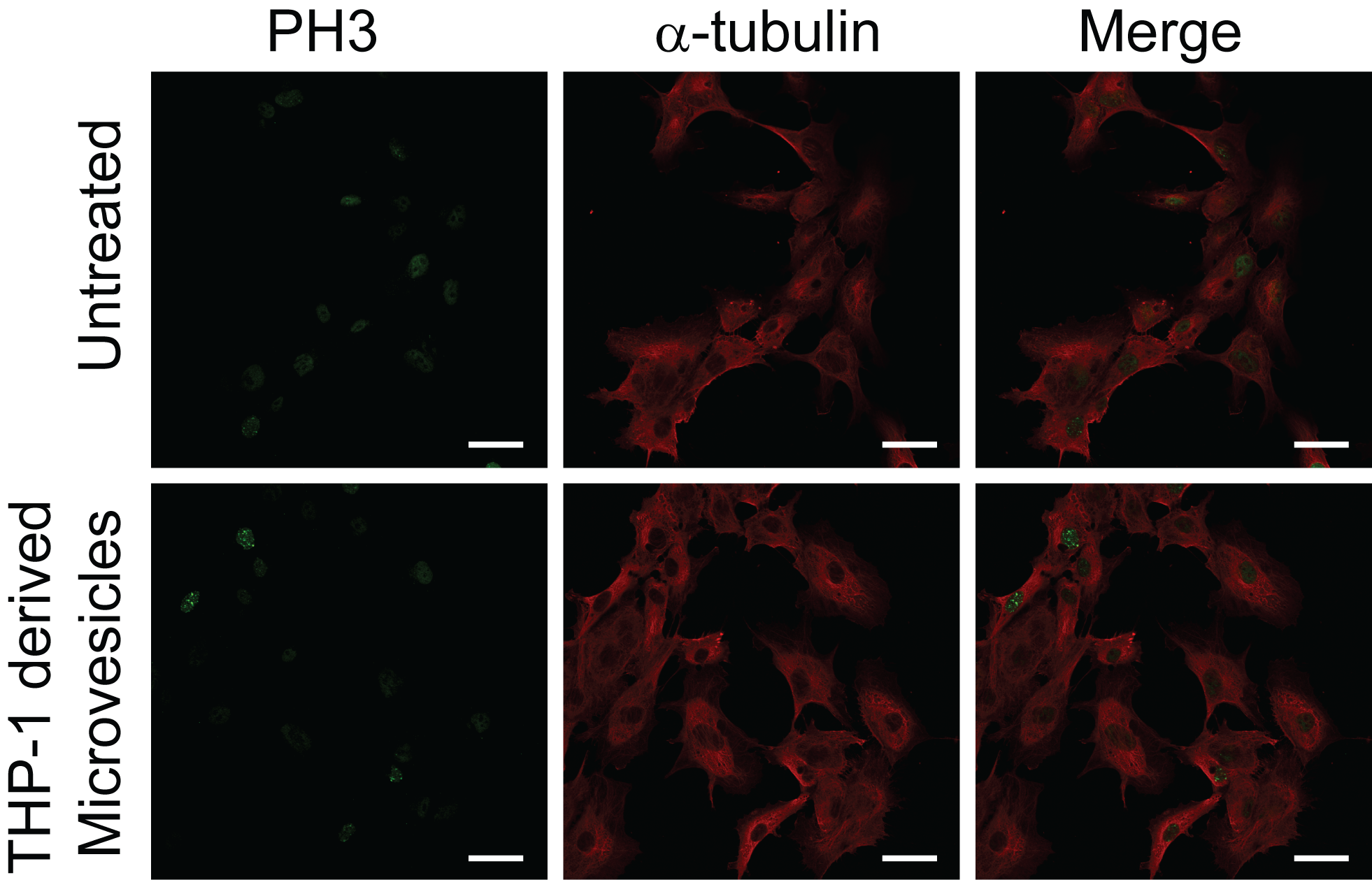

Supplement: Additional file 8 — The effect of THP-1-derived microvesicles on endothelial cell proliferation. After 12 hours, microvesicle-treated endothelial cells were immunostained with anti-phospho-histone H3 (green) and anti-α-antibodies (red). Scale bars represent 40 μm. [file 1471-2164-10-556-S8.DOC]
