# Supplementary material for: Colorectal cancer cell-derived microvesicles are enriched in cell cycle-related mRNAs that promote proliferation of endothelial cells
Source: BMC Genomics. 2009 Nov 25;10:556. doi: 10.1186/1471-2164-10-556 (PMC2788585; doi:10.1186/1471-2164-10-556)
Supplement: Additional file 9 — A subset of the 15 genes (CCNA2, CDKN3, CENPF, KIF23, and NEK2) whose expression is relatively enriched in CRC compared to other tissues and cells. Note that the 15 genes are those shared between 20 M-phase-related genes and 36 genes with differential expression patterns in patients with CRC. [file 1471-2164-10-556-S9.DOC]

**Additional file 9.** A subset of the 15 genes (*CCNA2*, *CDKN3*, *CENPF*, *KIF23*, and *NEK2*) whose expression is relatively enriched in CRC compared to other tissues and cells. Note that the 15 genes are those shared between 20 M-phase-related genes and 36 genes with differential expression patterns in patients with CRC

**
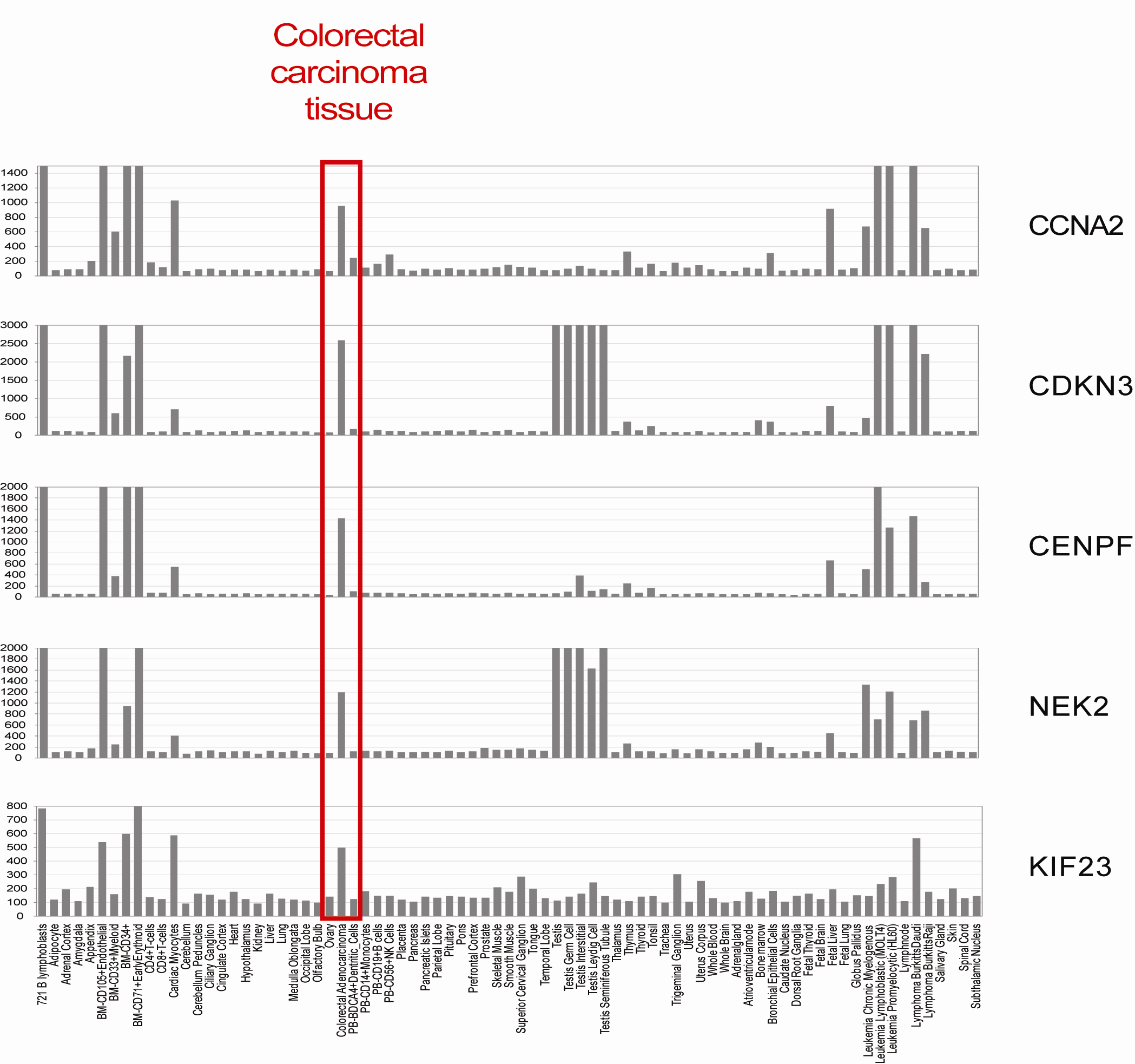
**
